# Ferroportin downregulation promotes cell proliferation by modulating the Nrf2–miR-17-5p axis in multiple myeloma

**DOI:** 10.1038/s41419-019-1854-0

**Published:** 2019-08-19

**Authors:** Yuanyuan Kong, Liangning Hu, Kang Lu, Yingcong Wang, Yongsheng Xie, Lu Gao, Guang Yang, Bingqian Xie, Wan He, Gege Chen, Huiqun Wu, Xiaosong Wu, Fenghuang Zhan, Jumei Shi

**Affiliations:** 1 Department of Hematology, Shanghai Tenth People’s Hospital, Tongji University Cancer Center, Tongji University School of Medicine, 200072 Shanghai, China; 2 Department of Internal Medicine, University of Iowa Carver College of Medicine, Iowa City, IA USA

**Keywords:** miRNAs, Myeloma

## Abstract

Recent findings demonstrate that aberrant downregulation of the iron-exporter protein, ferroportin (FPN1), is associated with poor prognosis and osteoclast differentiation in multiple myeloma (MM). Here, we show that FPN1 was downregulated in MM and that clustered regularly interspaced short palindromic repeat (CRISPR)-mediated FPN1 knockout promoted MM cell growth and survival. Using a microRNA target-scan algorithm, we identified miR-17-5p as an FPN1 regulator that promoted cell proliferation and cell cycle progression, and inhibited apoptosis—both in vitro and in vivo. miR-17-5p inhibited retarded tumor growth in a MM xenograft model. Moreover, restoring FPN1 expression at least partially abrogated the biological effects of miR-17-5p in MM cells. The cellular iron concentration regulated the expression of the iron-regulatory protein (IRP) via the 5′-untranslated region of IRP messenger RNA and modulated the post-transcriptional stability of FPN1. Bioinformatics analysis with subsequent chromatin immunoprecipitation-polymerase chain reaction and luciferase activity experiments revealed that the transcription factor Nrf2 drove FPN1 transcription through promoter binding and suppressed miR-17-5p (which also increased FPN1 expression). Nrf2-mediated FPN1 downregulation promoted intracellular iron accumulation and reactive oxygen species. Our study links FPN1 transcriptional and post-transcriptional regulation with MM cell growth and survival, and validates the prognostic value of FPN1 and its utility as a novel therapeutic target in MM.

## Introduction

Iron is an essential nutrient for various cellular processes, including DNA replication, cell-cycle progression, heme synthesis, and enzyme-mediated functions. Cellular iron homeostasis is regulated by a sophisticated system that responds to intracellular iron levels and a sophisticated gene network that maintains the intracellular storage, utilization, and export of iron^1^. Among these, ferroportin (FPN1; also known as SLC40A1) is a cell-surface transmembrane protein and the only known mammalian iron exporter for non-heme iron^2^. FPN1 is predominantly expressed on duodenal enterocytes, placental cells, hepatocytes, and reticuloendothelial macrophages^3–5^ and is an essential component for both cellular and systemic iron balance^6^. Hepcidin secreted by the liver binds to FPN1 and triggers FPN1 internalization and degradation, thereby blocking iron delivery to cells^7^. Thus, the hepcidin–FPN1 axis is predominantly responsible for systemic iron homeostasis.

Many cancers exhibit an increased need for intracellular iron and persistent iron stimulation can increase the risk for tumorigenesis^8^. Given the important iron-regulatory role of FPN1, its dysregulation may contribute to persistent iron stimulation^9^ and cancer development^10^. FPN1 expression is regulated at the messenger RNA (mRNA) level^11,12^, at the post-transcriptional level by the iron-regulatory protein/iron-responsive element (IRP/IRE) system^13,14^, and post-translationally by the organismal iron status via the peptide hormone, hepcidin^7^. Iron released from hemoglobin regulates iron exporter FPN1 translation, involving the IRE within the FPN1 5′-untranslated region (UTR). FPN1 transcripts contain an IRE sequence in the 5′-UTR, which binds IRP proteins during iron deprivation, causing translational efficiency inhibition at the post-transcriptional level^15^. Many previous reports have revealed that FPN1 mutations and intron polymorphisms lead to hyperferritinemia, inflammatory reactions, and several cancers^16–18^. However, the biological effects of abnormal FPN1 expression on tumor behaviors and the molecular mechanisms underlying FPN1 dysregulation in multiple myeloma (MM) cells remain largely unexplored.

MicroRNAs (miRNAs) represent a class of small non-coding RNAs, 18–25 nucleotides long, which negatively regulate gene expression at the post-transcriptional level by binding the 3′-UTR of target mRNAs^19^, leading to translation repression^20^ and/or mRNA degradation^3^. Recently, aberrant miRNAs were screened to examine their roles in mediating biological processes in MM, such as cell proliferation, migration, invasion, apoptosis, drug resistance, and signal transduction^21,22^. Patients with high miR-17, miR-20, and miR-92 expression had shorter progression-free survival (PFS)^23^, and the myc-inducible miR-17-92 miRNA cluster participated in MM tumorigenesis and tumor progression^24^. The recently developed pharmacological tool MIR17PTi, a first-in-class inhibitor of pri-miR-17-92, can trigger apoptosis by impairing homeostatic myc–miR-17-92 feed-forward loops, with advantageous safety and pharmacokinetics profiles in MM^25^.

The transcription factor, nuclear factor (erythroid-derived 2)-like 2 (Nrf2), mediates oxidative and electrophilic stress, and regulates the expression of several genes involved in iron metabolism^26^. Recent studies showed that the cellular liable iron pool (LIP) was mediated by several genes involved in iron storage and iron export, which was controlled by Nrf2. In addition, Nrf2 can promote FPN1 expression and a positive correlation was found between Nrf2 and FPN1 expression in prostate and breast cancers^27,28^. However, Nrf2 suppressed FPN1 transcription in ovarian cancer cells^29^. These findings suggest that the specific control of FPN1 by Nrf2 is of great importance, although much work needs to be done to understand the regulation.

MM is a plasma cell neoplasm. Despite the development of novel therapeutic agents, MM remains incurable. In this study, we explored the potential roles of the transcription factor Nrf2 and miR-17-5p in transcriptional and post-transcriptional FPN1 regulation. We demonstrated that clustered regularly interspaced short palindromic repeat (CRISPR)-mediated FPN1 knockout in MM cells promoted LIP and reactive oxygen species (ROS) accumulation, which are important for malignant cell growth and survival. These results provide a mechanistic explanation between iron metabolism and MM.

## Results

### MiR-17-5p promoted cell proliferation and cell-cycle progression, and inhibited apoptosis in MM in vitro and in vivo

**Fig. 1:**
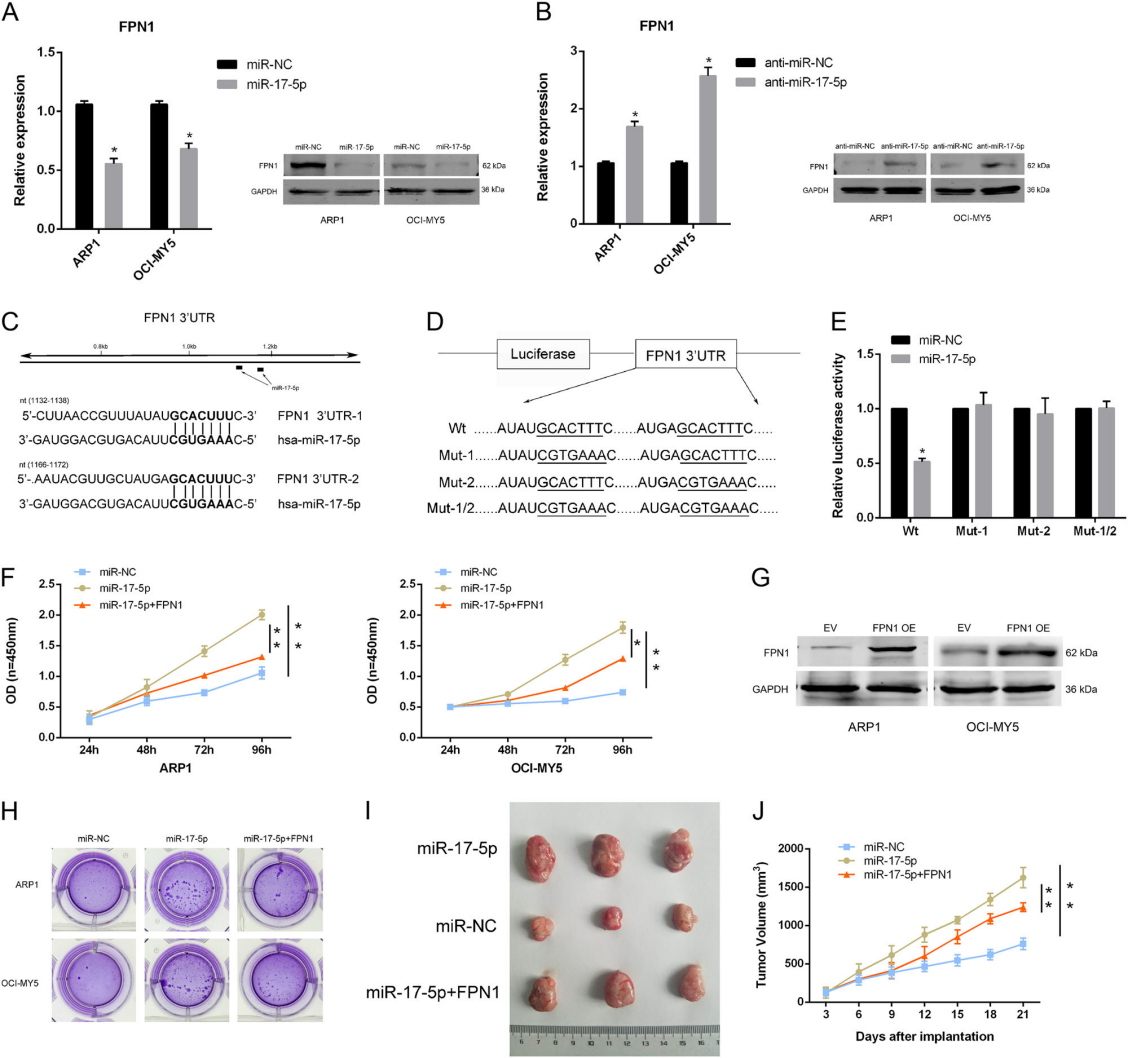
miR-17-5p targets FPN1 and restoring expression of FPN1 suppresses the miR-17-5p-induced effects in MM cells. **a**, **b** FPN1 expression at the mRNA and protein level in indicated cell lines was determined by qRT-PCR and western blot. **c** Diagram of predicted binding sites of miR-17-5p on the 3′-UTR of the *FPN1* gene. **d** Diagram of FPN1 3′-UTR wild-type and mutant reporter constructs. **e** Luciferase reporter assays were performed in HEK293T cell with co-transfection of indicated wild-type or mutant 3′-UTR constructs and miR-17-5p mimic. **f**, **h** Cell proliferation and colony formation were assessed in myeloma cells transfected with miR-17-5p mimic or miR-NC, with or without plasmid pCDH-FPN1 vector. **g** FPN1 expression was identified in FPN1 overexpressing multiple myeloma cells by western blot. **i** Tumor samples were collected and images were captured using a digital camera. **j** The indicated cells were injected into the upper flank region of nude mice (*n* = 3 per group). Tumor sizes were measured every 3 days using a caliper. Data are expressed as the mean ± standard deviation (*n* = 3). *p < 0.05, **p < 0.01

To investigate whether miRNAs are associated with aberrant FPN1 expression and MM progression, online miRNA target-prediction tools were used to generate FPN1-targeting miRNA candidates. Global miRNA-profiling data showed that miR-17 was associated with PFS and overall survival (OS)^30^. miR-17 was also positively associated with the patients’ gene-expression profiling (GEP)-defined risk scores and cell proliferation indexes^31^. We found that the 3′-UTR of FPN1 mRNA contains two putative miR-17-5p-binding sites (Fig. 1c) and the human and mouse transcripts share highly conserved seed sequences; thus, miR-17 was investigated further.

**Fig. 2:**
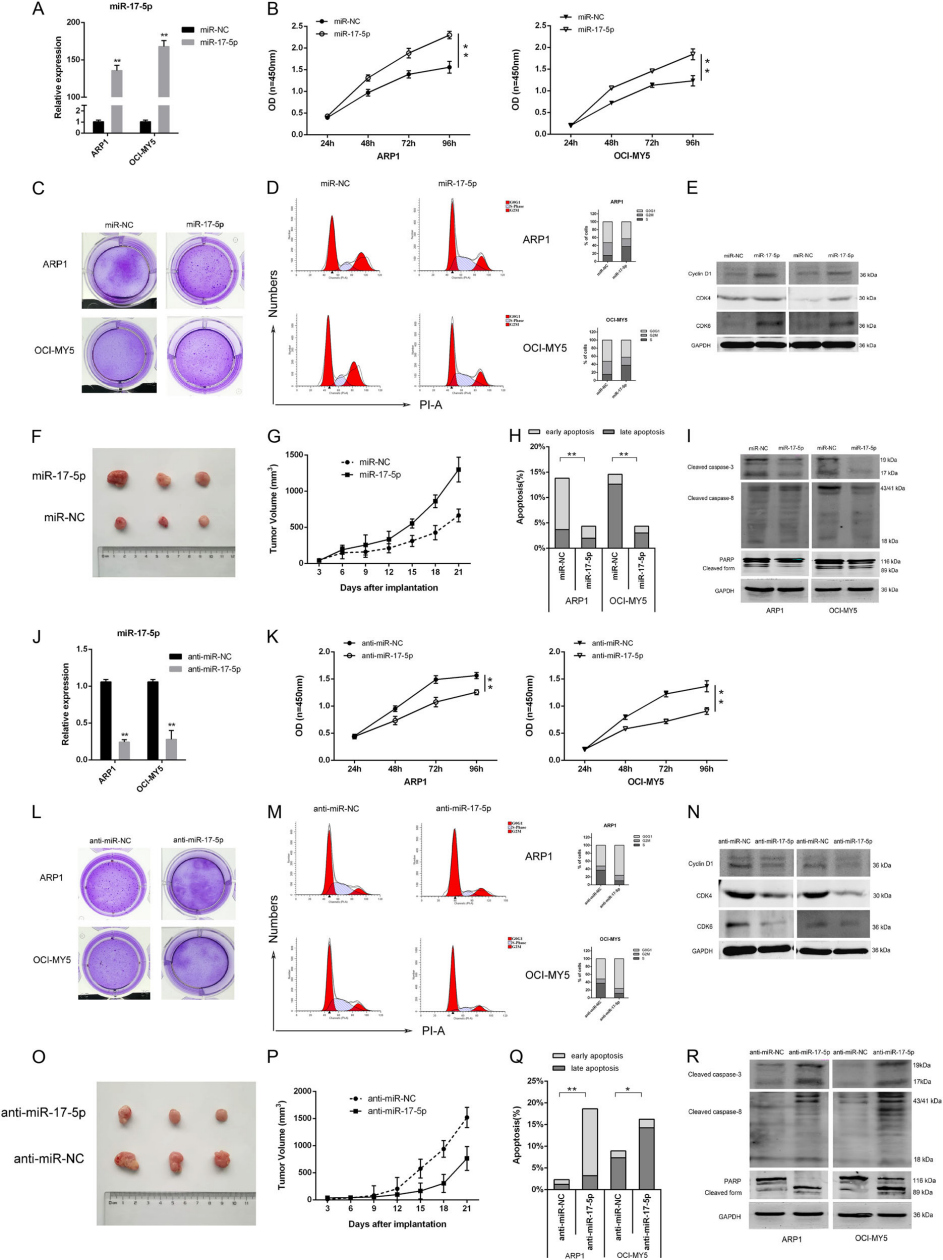
miR-17-5p promoted MM cell proliferation, colony formation, cell cycle progression, and inhibited cell apoptosis in vitro and in vivo. **a** The total RNA was isolated from ARP1 and OCI-MY5 cells and the level of miR-17-5p mRNA were determined by qRT-PCR after transfection of control and miR-17-5p mimic. Overexpression of miR-17-5p promoted cell proliferation **b**, colony formation **c**, cell cycle progression **d**, and inhibited apoptosis **h**, while miR-17-5p knockdown was assessed by qRT-PCR **j** and inhibited cell proliferation **k**, colony formation **l**, cell cycle progression **m**, and promoted apoptosis **q** in myeloma cells. Western blot analysis of cell-cycle regulator proteins Cyclin D1, CDK4, and CDK6 **e**, **n**, apoptosis-related protein expression **i**, **r** in the presence and absence of miR-17-5p. Representative pictures of MM xenografts from both ARP1-miR-17-5p **f** and ARP1-anti-miR-17-5p cells **o**. Tumor growth curve revealing that miR-17-5p overexpression significantly promotes growth **g**, while miR-17-5p knockdown inhibits tumor growth in vivo. *p < 0.05; **p < 0.01

ARP1 and OCI-MY5 cells were transfected with either a miR-17-5p mimic or inhibitor, and the efficacies of miR-17-5p overexpression ((ARP1: 135.703 fold NC ± 12.048, *p* < 0.001; OCI-MY5: 167.901 fold NC ± 13.667, *p* < 0.001) and knockdown (ARP1: 0.242-fold NC ± 0.057, *p* < 0.001; OCI-MY5: 0.279-fold NC ± 0.206, *p* = 0.003) were verified by quantitative real-time polymerase chain reaction (qRT-PCR) analysis (Fig. 2, a). Next, the effects of miR-17-5p on proliferation, colony formation, cell-cycle progression, and apoptosis were assessed. The miR-17-5p mimic increased cell growth (Fig. 2b) and colony formation (Fig. 2c). Conversely, miR-17-5p knockdown repressed MM cell proliferation and colony formation (Fig. 2, k). Flow-cytometric analysis revealed that miR-17-5-p overexpression induced cell-cycle progression from G1 to S phase (miR-NC, 52.705% ± 1.497 in G1 phase; miR-17-5p, 42.732% ± 2.367; Fig. 2d) and that miR-17-5p inhibition arrested MM cells in G0/G1 phase (miR-NC, 52.402% ± 0.764; miR-17-5p, 75.685% ± 1.043; Fig. 2m). Accordingly, cell-cycle proteins (cyclin D1 and CDK4/6) were upregulated in miR-17-5p-overexpressing MM cells (Fig. 2, e). We also found that the miR-17-5p mimic inhibited apoptosis (miR-NC, 13.813% ± 0.600; miR-17-5p, 4.346% ± 1.597, *p* < 0.001; Fig. 2h), whereas the miR-17-5p inhibitor reversed this trend (miR-NC, 2.306% ± 0.275; miR-17-5p, 18.643% ± 3.287, *p* = 0.001; Fig. 2q). Consistently, western blotting showed that miR-17-5p inhibitors activated the expression of apoptosis related-proteins in MM cells (Fig. 2, i).

Next, we measured tumor growth in a nude xenograft mouse model. After a 3-week inoculation, the mice were sacrificed, and isolated tumor tissues were imaged. Tumor sizes and volumes in the miR-17-5p group were much larger than those in the control group (Fig. 2, f). Conversely, miR-17-5p knockdown retarded xenograft tumor growth (Fig. 2, o).

### miR-17-5p directly targeted FPN1 in MM cells

Bioinformatics analysis predicted that miR-17-5p binds the FPN1 3′-UTR. By qRT-PCR and western blot analysis, we found that miR-17-5p overexpression significantly reduced the mRNA-expression (ARP1: 0.555-fold NC ± 0.075, *p* < 0.001; OCI-MY5: 0.682 fold NC ± 0.079, *p* = 0.002) and protein-expression levels of FPN1 (Fig. 1a), whereas miR-17-5p inhibitors noticeably increased FPN1 expression in ARP1 (1.688 fold NC ± 0.154, *p* = 0.002) and OCI-MY5 (2.573-fold NC ± 0.255, *p* < 0.001) myeloma cells (Fig. 1b).

Next, we constructed luciferase reporters by cloning the 3′-UTR of FPN1 (and derived mutants) downstream of the *Renilla* luciferase gene in the psiCHECK2 vector. HEK293T cells were co-transfected with vectors harboring the wild-type or mutant FPN1 3′-UTR (Fig. 1d) and the miR-17-5p mimic. Luciferase activity markedly decreased (0.516 ± 0.030, *p* < 0.001) after co-transfection with the miR-17-5p mimic and wild-type reporters, but was barely affected the double mutants (Fig. 1e), suggesting that miR-17-5p targeted both FPN1-binding sites.

We also transfected miR-17-5p-overexpressing myeloma cells with a FPN1-expression vector (Fig. 1g). FPN1 restoration partially abrogated the effect of miR-17-5p, resulting in decreased proliferation of miR-17-expressing cells (Fig. 1f) and reduced colony formation (Fig. 1h). An in vivo xenograft mouse model showed that the average tumor size and volume decreased after FPN1 overexpression in ARP1/miR-17-5p cells (Fig. 1, i).

### Nrf2 regulated FPN1 expression via miR-17-5p

**Fig. 3:**
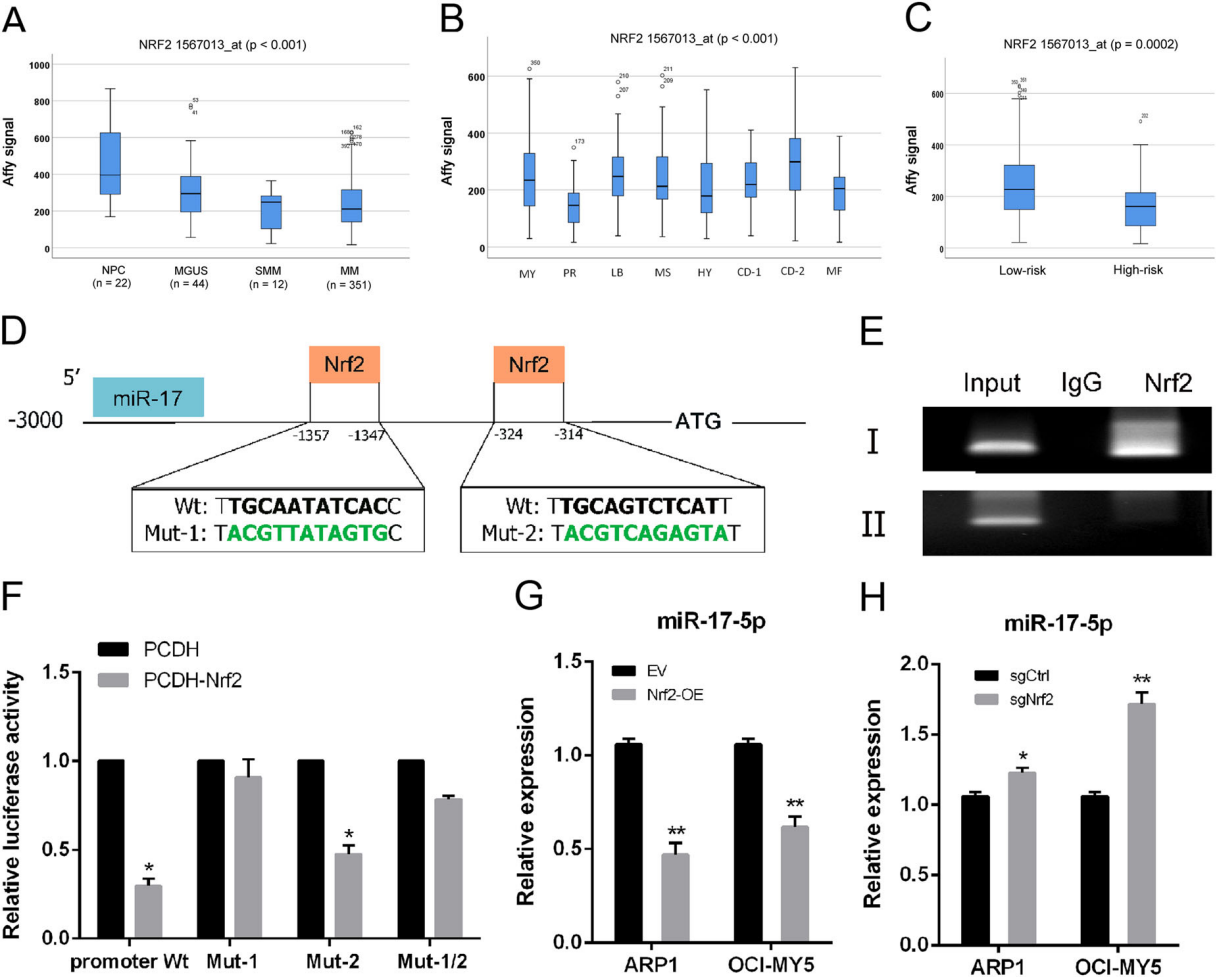
Nrf2 regulates FPN1 expression via miR-17-5p. **a** Bar plots depict the Affymetrix signal of FPN1 in NPCs, MGUS, smoldering myeloma (SMM), and newly diagnosed multiple myeloma (MM; TT2 cohort). One-way ANOVA was performed and identified the *p* < 0.001 among these four groups. **b** The expression of FPN1 among eight multiple myeloma subgroups is highly variable (*p* < 0.001 by one-way ANOVA). **c** The expression of FPN1 between low-risk and high-risk subgroups in multiple myeloma. The difference was compared by the Student's *t-*test between these two groups. **d** Two predicted Nrf2-binding sites in the promoter of miR-17-5p. **e** ChIP assays showing that Nrf2 can bind to potential binding sites in the miR-17-5p promoter. **f** Relative luciferase activity of the indicated promoter vectors in HEK293T cells transfected with Renilla luciferase plasmids. **g**, **h** miR-17-5p expression was detected by qRT-PCR in the indicated cell lines

Next, we investigated transcription factor Nrf2 expression in primary MM cells. Nrf2 transcript levels were significantly lower in patients with MM, compared with the corresponding levels in patients with monoclonal gammopathy of undetermined significance (MGUS) and in healthy donors (*p* < 0.001; Fig. 3a). Nrf2 expression was lowest in the proliferation subgroup (PR), which represents patients with the poorest prognosis (*p* < 0.001; Fig. 3b) and was significantly decreased in high-risk myeloma samples (*p* = 0.0002; Fig. 3c).

JASPAR bioinformatics software was utilized to analyze 3000 bases upstream of the transcriptional start site of the precursor, miR-17-5p. We designed primer pairs to amplify two promoter regions upstream of miR-17-5p (Fig. 3d) and performed chromatin immunoprecipitation (ChIP)-PCR assay in MM cells. ChIP assays with a Nrf2 antibody showed that Nrf2 had greater occupancy in the upstream region (–1357 to –1347) of miR-17-5p (Fig. 3e). Moreover, luciferase activities decreased significantly when conserved binding-site 2 was mutated (0.476 ± 0.049, *p* < 0.001), whereas mutating binding-site 1 nearly rescued the decrease (Fig. 3f). In agreement, Nrf2 overexpression in ARP1 and OCI-MY5 cells decreased miR-17-5p expression (ARP1: 0.471 ± 0.108, *p* = 0.001; OCI-MY5: 0.618 ± 0.099, *p* = 0.002) in parental empty vector cells (Fig. 3g). Conversely, knocking out Nrf2 in ARP1 and OCI-MY5 cells increased miR-17-5p expression (ARP1: 1.228 ± 0.061, *p* = 0.022; OCI-MY5: 1.716 ± 0.143, *p* = 0.001) in MM-sgNrf2 cells (Fig. 3h).

### Nrf2 transactivation regulated FPN1 expression

**Fig. 4:**
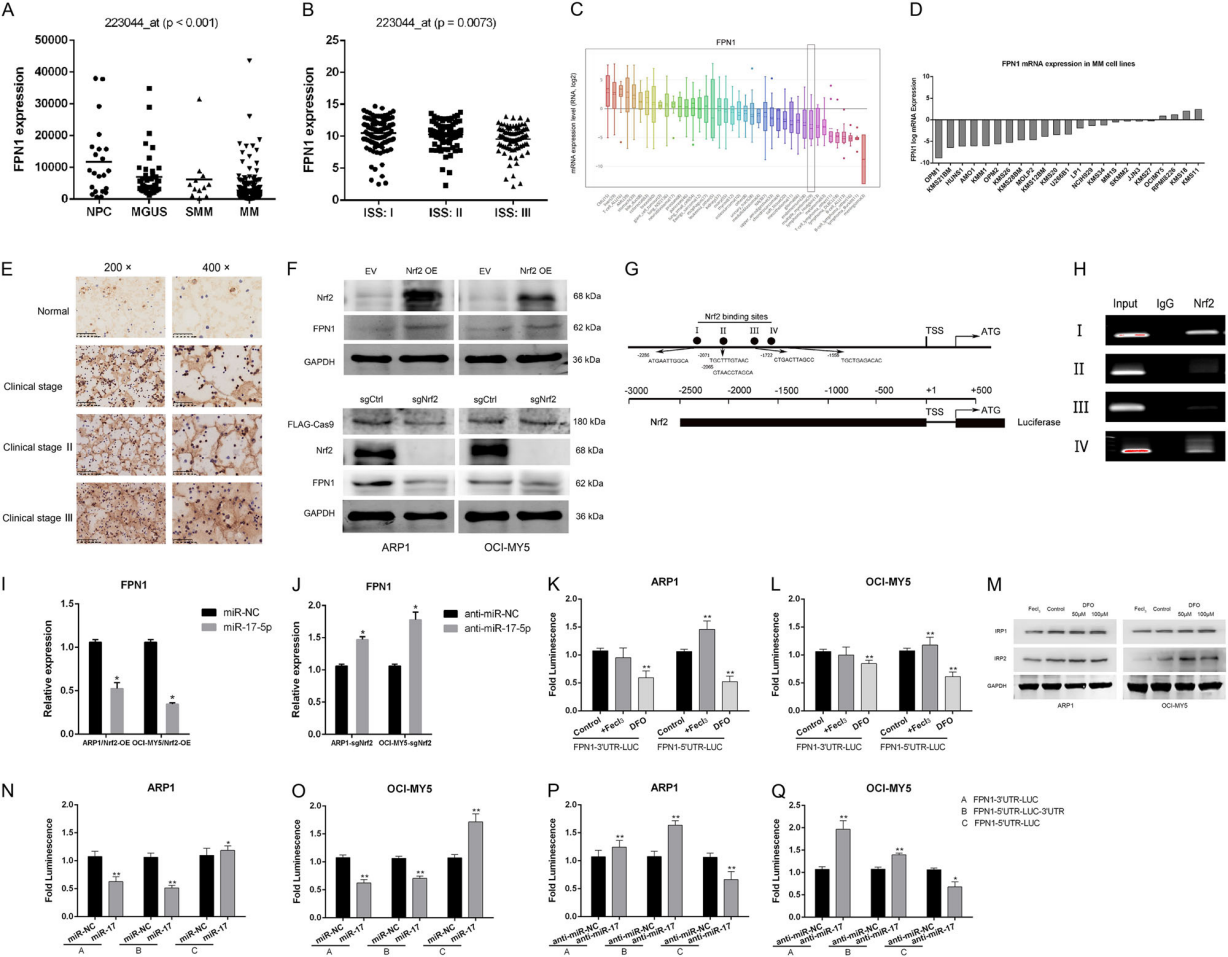
Nrf2 transactivated regulates FPN1 and IRP-mediated 5′-UTR regulation. **a** FPN1 mRNA expression was analyzed in MM patient samples using publicly available data sets (GSE2658 and GSE5900). Dot-plots present the expression of FPN1 in NPC, MGUS, SMM, and newly diagnosed multiple myeloma (MM; TT2 cohort); analysis of variance (ANOVA) followed by Dunnett’s test. **b** FPN1 level was compared between MM patient samples of different ISS stage (GSE19784). **c** FPN1 expression in differential human malignancies from CCLE database. MM tissues expressed relatively lower FPN1 compared with most of the other human cancers in a reliable confidence interval. **d** FPN1 expression in different MM cell lines from CCLE database. FPN1 mRNA levels are commonly low in various MM cell lines. **e** Immunohistochemical analysis of ferroportin localization in human multiple myeloma tissues compared with normal bone marrow (left panel: magnification ×200; right panel: magnification ×400). **f** Nrf2 and FPN1 expression levels were detected by western blot in indicated cell lines. **g** Bioinformatic analysis of potential Nrf2-binding sites in the FPN1 promoter using an online software JASPAR. **h** The interaction of Nrf2 with the FPN1 promoter was assessed by CHIP analysis. **i**, **j** FPN1 expression was detected by qRT-PCR in the indicated cell lines. **k**, **l** Relative luciferase activity of the FPN1 3′UTR and 5′UTR luciferase reporter constructs transfected in ARP1 and OCI-MY5 cells following iron-supplementation (FeCl_3_) or iron-depletion (DFO) were shown when compared with untreated condition. **m** Western blot analysis of IRP1 and IRP2 protein levels in the iron-rich and iron-deficient conditions. **n**–**q** Activity of FPN 3′UTR, FPN-5′UTR-LUC-3′UTR, and FPN 5′UTR reporter following treatment with miR-17-5p mimic or inhibitor. Data are presented as the mean ± SD of three independent experiments (*n* = 3). *p < 0.05, **p < 0.01

We investigated FPN1 expression in primary MM cells using publicly available data^32–34^. FPN1 expression was examined by GEP in plasma cells from 22 healthy donors, 44 patients with MGUS, 12 patients with smoldering myeloma, and 351 patients newly diagnosed with MM. FPN1 expression was strongly dysregulated in MM cells versus healthy donor plasma cells (*p* < 0.001; Fig. 4a) and closely correlated to International Staging System classifications (*p* = 0.0073; Fig. 4b). We then searched the Cancer Cell Line Encyclopedia online database to evaluate the expression status of FPN1. FPN1 expression was markedly reduced in 40 tumor specimens, and FPN1 levels in differential MM cell lines were frequently low compared to their relatively normal controls (Fig. 4, c). Immunohistochemical analysis verified that excess FPN1 proteins deposited in the cell membranes in bone marrow smears, causing a darker background color versus non-MM cells, indicating that increased FPN1 membrane localization correlated with the clinical stage (Fig. 4e).

The mechanism of FPN1 mRNA regulation in cancer is just beginning to emerge. We found that Nrf2 overexpression increased FPN1 protein levels, whereas Nrf2 knockout significantly downregulated FPN1 protein in ARP1 and OCI-MY5 cells (Fig. 4f). A 3-kb region of the human FPN1 promoter was cloned to identify a potential transcription factor-binding site. Moreover, JASPAR analysis predicted that several Nrf2-binding motifs may be present inside the putative FPN1 promoter region, referred to as sites I–IV (Fig. 4g). We confirmed Nrf2 binding to the FPN1 promoter in ChIP assays (nucleotide positions –2286 to –2276 and –1556 to –1546; Fig. 4h). In addition, miR-17-5p overexpression blocked FPN1 upregulation induced by Nrf2 overexpression (Fig. 4i), and miR-17-5p knockdown blocked FPN1 downregulation induced by Nrf2 knockout in myeloma cells (Fig. 4j).

### Both IRP-mediated 5′-UTR targeting and miR-17-5p-mediated 3′-UTR targeting post-transcriptionally regulated FPN1 expression

To investigate IRP-mediated regulation of FPN1 expression, we constructed a luciferase reporter by cloning the FPN1 5′-UTR upstream of the luciferase gene. ARP1 and OCI-MY5 cells were transfected with vectors harboring the FPN1 5′-UTR or 3′-UTR under iron-replete and iron-deficient conditions, using FeCl_3_ and the iron chelator deferoxamine (DFO), respectively. DFO significantly inhibited FPN1 3′-UTR luciferase-reporter activity (0.590 ± 0.249, *p* = 0.011), whereas iron supplementation did not affect FPN1 3′-UTR-reporter activity in ARP1 cells (0.951 ± 0.345, *p* > 0.05; Fig. 4k). Similar results were seen in OCI-MY5 cells (Fig. 4l). FPN1 5′-UTR-luciferase activity significantly increased (1.459 ± 0.301, *p* < 0.05) during iron supplementation and significantly decreased (0.521 ± 0.197, *p* = 0.002) during iron deprivation. Similar results were seen in OCI-MY5 cell line. We examined IRP1 and IRP2 expression in MM cells treated with 50 μM FeCl_3_ or DFO (50 μM, 100 μM) for 48 h. Western blot analysis demonstrated that DFO increased IRP2 expression, but not IRP1 expression (Fig. 4m).

Finally, we constructed a luciferase reporter (FPN1-5′UTR-LUC-3′UTR) by cloning the 5′-UTR and 3′-UTR of FPN1 upstream and downstream of the *Renilla* luciferase gene, respectively, in the psiCHECK2 vector to mimic endogenous FPN1 mRNA expression. miR-17-5p overexpression decreased FPN1 3′UTR luciferase activity to 30.8% (±0.083) and 27.9% (±0.122) of the control in ARP1 and OCI-MY5 cells, and FPN1-5′UTR-LUC-3′UTR activity to 52.3% (±0.047) and 27.8% (±0.072) of the control respectively (Fig. 4n, o). miR-17-5p overexpression significantly increased FPN1 5′UTR-luciferase activity in both cells. Conversely, miR-17-5p knockdown in myeloma cells increased FPN1 3′-UTR (1.299 ± 0.117 and 2.382 ± 0.377, *p* < 0.01) and FPN1-5'UTR-LUC-3'UTR (1.613 ± 0.082 and 1.462 ± 0.071, *p* < 0.01) luciferase activities, versus the inhibitor control (Fig. 4, p). After miR-17-5p inhibition, 5′-UTR of FPN1 activity decreased in both ARP-1 (0.464 ± 0.141, *p* = 0.002) and OCI-MY5 (0.661 ± 0.232, *p* < 0.01) cells.

### Low FPN1 conferred MM cell growth and survival

**Fig. 5:**
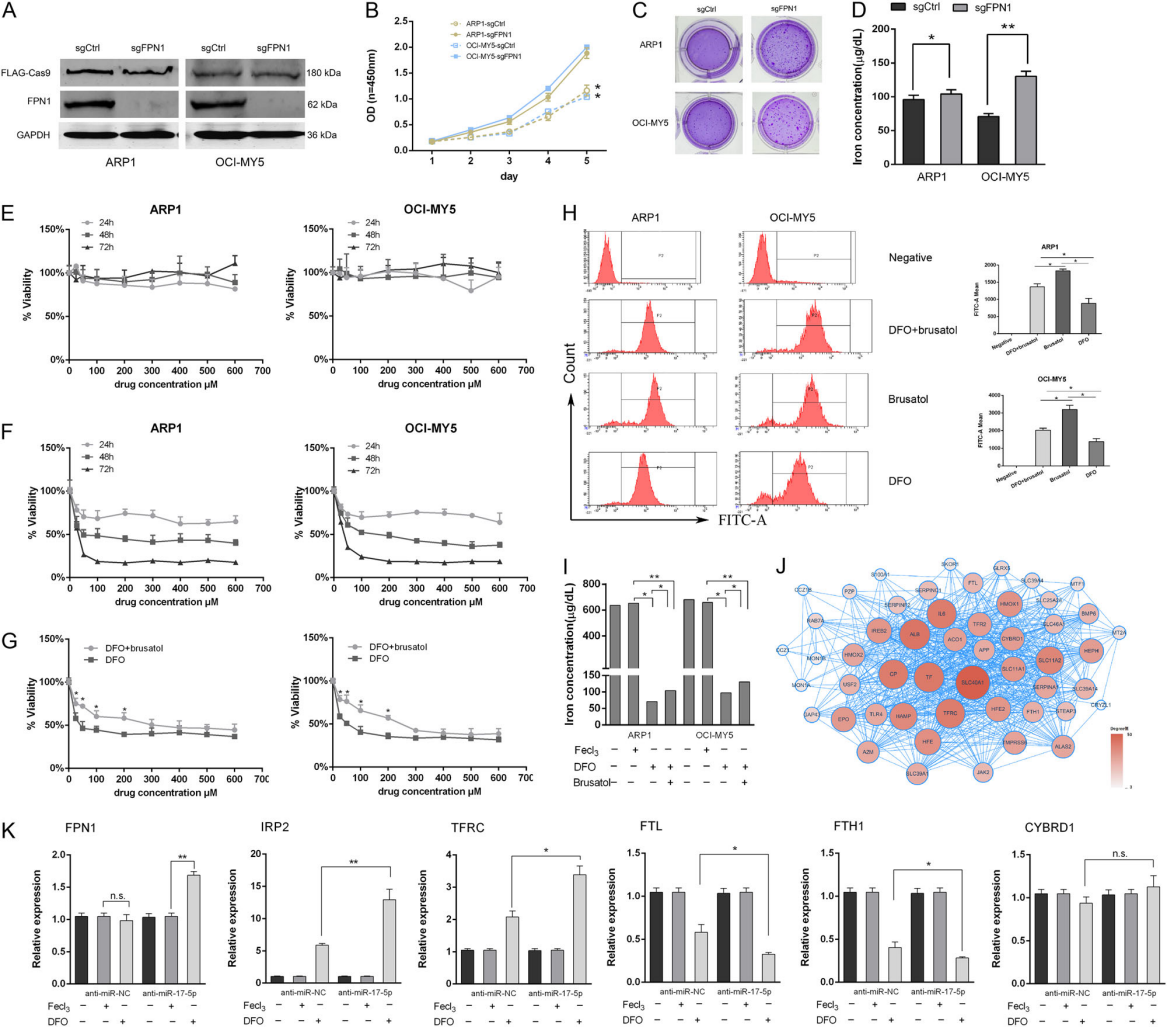
FPN1 regulates multiple myeloma cell intracellular iron and ROS. **a** ARP1 and OCI-MY5 cells expressing Cas9 were transduced with sgRNA against FPN1 (sgFPN1) or control sgRNA (sgCtrl). Western blot confirmed FPN1 knockout. **b** FPN1-sgRNA MM cell lines ARP1, OCI-MY5 as well as their controls were analyzed using a Cell Counting kit-8 assay for consecutive 5 days for cell growth. **c** Clonogenic ability of myeloma cells was examined after FPN1 knockout. **d**, **i** The intracellular iron was measured with or without different reagents (50 μM DFO and 5 μM brusatol) in indicated cells using QuantiChrom^TM^ Iron Assay Kit. **e**, **f** Two myeloma cells were treated with deferoxamine (DFO) and FeCl_3_ at indicated concentration for 24, 48, and 72 h, and viability was then analyzed. **g** FPN1-sgRNA MM cell lines ARP1, OCI-MY5 were treated for 48 h with varying concentrations of DFO and/or brusatol. **h** ARP1 and OCI-LY5 FPN1-knockout cells with different treatment (50 μM DFO and 5 μM brusatol) for 48 h in indicated cells and stained with H_2_DCFDA; then the level of ROS was detected by flow cytometry and data are presented as the mean fluorescence intensity. **j** Bioinformatics analysis indicated that most interact proteins of FPN1 are involved in iron metabolism. **k** qRT-PCR was used to examine expression of genes related to iron metabolism. Data are expressed as the means ± standard deviation (*n* = 3). *p < 0.05, **p < 0.01, n.s., not significant, compared to the vehicle control group; Student’s *t*-test

Decreased FPN1 expression in MM-patient samples correlated with short event-free survival (EFS) and inferior OS, with poor patient outcomes in clinical trials^35^. Here, we introduced a single guide (sg) RNA targeting FPN1 into MM cell lines stably expressing *Cas9*, and FPN1-protein levels were verified by western blotting (Fig. 5a). CRISPR-mediated FPN1 knockout promoted cell growth (Fig. 5b) and increased MM-cell colony formation (Fig. 5c).

DFO was used to examine the role of iron in the contributions of FPN1 to cell growth and transformation. DFO reduced myeloma cell viability in a time-dependent manner, but not in a dose-dependent manner (Fig. 5f). FeCl_3_ showed minimal cytotoxicity in both cell lines, at concentrations up to 600 µM (Fig. 5e).

### Both Nrf2-mediated transcriptional regulation and miR-17-5p-mediated FPN 3′-UTR regulation altered cellular iron and ROS levels

Knocking out FPN1 in ARP1 and OCI-MY5 cells resulted in higher intracellular iron levels, compared with sgCtrl treatment (Fig. 5d). Treatment with DFO alone significantly inhibited cell proliferation and reduced the intracellular iron level, whereas combined treatment with DFO and the Nrf2 inhibitor brusatol partially promoted proliferation and increased intracellular iron in both FPN1-knockout cell lines (Fig. 5, g).

Recent data have shown that a higher intracellular iron level and metabolic rate may induce intracellular ROS generation, causing oxidative stress-mediated gene mutations and constitutive activation of tumorigenesis-related signaling pathways^36,37^. We evaluated ROS levels after DFO and brusatol treatment in FPN1-knockout cells by flow cytometry using the specific, oxidation-sensitive, fluorescent dye H_2_DCFDA. ARP1 and OCI-MY5 FPN1-knockout cells showed relatively higher ROS levels, compared with DFO or brusatol treatment alone (Fig. 5h). In addition, DFO treatment markedly reduced the mean H_2_DCFDA fluorescence intensity across all four tested groups.

Previous data identified FPN1 as the only known mammalian iron exporter from the cytosol to the extracellular milieu^38^. Most FNP1-interacting proteins are closely related to iron metabolism (http://string-db.org) (Fig. 5j). Specifically inhibiting miR-17-5p activity in ARP1 cells significantly and reproducibly increased FPN1-protein levels following iron depletion (Fig. 5k). Cells with lost miR-17-5p function showed a greater degree of iron deficiency, as evidenced by increased protein levels of IRP2, increased transferrin receptor (TFRC) mRNA expression, and decreased ferritin light chain (FTL) and ferritin heavy chain 1 (FTH1) mRNA expression. However, cytochrome b reductase 1 (CYBRD1) expression did not significantly change.

## Discussion

Iron is an essential nutrient for all cells and iron-regulatory proteins play critical roles in diverse intracellular processes, including cell cycle progression, DNA synthesis, and metabolism. However, excess labile iron facilitates cancer development because it acts as a cofactor for proteins essential for sustaining growth and proliferation^39^. Modulation of iron-containing proteins (including FPN1 and ferratin proteins) is implicated in cancer. FPN1, a transmembrane protein, is overexpressed in many cancers and has proven crucial for cell proliferation and metastasis^18,35,39^. Ferritin, an intracellular iron-storage protein, maintains an appropriately sized labile iron pool for biosynthetic processes. Upstream signaling responsible for decreased FPN1-dependent iron export has remained elusive, especially in MM.

Here, we focused on a marked reduction of FPN1 expression at the basolateral membranes of enterocytes, hepatocytes, and macrophages of the reticuloendothelial system^5,7^. Previous data showed that the FPN1 protein was markedly decreased in breast cancer epithelial cells, and low FPN1 expression correlated with increased labile iron, which negatively correlated with clinic outcomes^39^. Moreover, reduced FPN1 expression was linked to poor survival and served as a strong and independent prognostic marker for MM^35^. Here, we downloaded datasets for MM patients and GEP data from the NCBI GEO database, observing that FPN1 was strongly dysregulated in MM samples versus healthy subjects. We also found that FPN1 expression decreased markedly in 40 tumor samples studied and that FPN1 levels in differential MM cells were commonly low. Immunohistochemical analysis also verified accumulating membrane localization of FPN1 with increasing malignant stages, consistent with previous reports showing that FPN1 expression was dysregulated in many types of cancers^40–42^. Moreover, FPN1 has been associated with short EFS and poor survival, and could be a strong and independent marker of poor prognosis in MM^35^.

Previous findings revealed that FPN1 levels are regulated by post-translational, post-transcriptional, and transcriptional regulatory mechanisms. Here, we sought to determine whether the FPN1–3′-UTR is an important regulatory region and whether miR-17-5p functions as a post-transcriptional regulator by directly targeting FPN1. miR-17-5p expression was significantly higher in MM patients than in healthy donors. Previous reports also showed that increased miR-17-5p expression associated positively with risk stratification and conferred a poor prognosis for MM patients^30^. We found that miR-17-5p overexpression promoted cell proliferation, colony formation, cell-cycle progression, and apoptosis inhibition, whereas miR-17-5p knockdown shows opposite trends with myeloma cells, in vitro. We then confirmed that FPN1 is a direct target of miR-17-5p in luciferase-activity assays and that FPN1 expression correlated inversely with miR-17-5p expression. Moreover, FPN1 restoration abrogated the effects of miR-17-5p on proliferation and colony formation, both in vitro and in vivo. Consistently, the miR-17-92 cluster miRNAs (miR-17 and miR-20a) were implicated in solid and hematologic tumors, with high miR-17-5p-expression levels correlating with poor clinical outcomes and tumorigenesis^24,43^.

Post-transcriptional, miRNA-mediated regulation of FPN1 translation was documented above, although the upstream modulation of FPN1-mRNA expression remains to be explored, especially in MM. Combinatorial regulation of RNA elements with binding proteins plays a key biological role in vertebrates and bacteria. The IRE-motif sequence, present in the 5′-UTR, shows 100% conservation between humans, mice, and rats^4^. With a putative IRE-binding IRP, FPN1 is an aconitase homolog. Iron-binding proteins (such as ferritin) can inhibit protein translation and sequester iron in the absence of intracellular iron^15^, DMT1, and TfR for iron uptake. In the case of FPN1, both the IRP-mediated 5′-UTR interaction may be further stabilized by the miR-17-5p-guided 3′-UTR to enable more dynamic and fine-tuned expression under a broad range of iron conditions. Several studies have demonstrated that Nrf2 induces FPN expression by binding to Maf-recognition element (MARE)/antioxidant-response element (ARE)-enhancer sequences in the FPN promoter in mouse macrophages^12,44^. However, controversy remains regarding the interaction between Nrf2 and FPN1. Xue et al.^27^ and Chen et al.^28^ demonstrated that Nrf2 promoted FPN1 expression and showed a positive correlation between these two proteins in prostate and breast cancers. Nevertheless, Nrf2 suppressed the iron export-related gene FPN1 in ovarian cancer cells^29^. In our study, ChIP experiments and dual-luciferase-reporter assays revealed that Nrf2 directly transactivated FPN1 expression and inhibited miR-17-5p expression in MM, indicating that Nrf2 can modulate FPN1 levels directly or indirectly through miR-17-5p. Thus, the underlying regulatory mechanism in regulating FPN1 expression requires further study.

An interesting question is how the modulation of FPN1 levels affect myeloma cell growth and survival. We observed that CRISPR-mediated FPN1 knockout significantly increased ARP1 and OCI-MY5 cell proliferation. Conversely, FPN1 overexpression inhibited cell proliferation, as previously reported^35^, confirming the role of FPN1 in MM cell growth. In addition, manipulating FPN1 expression promoted LIP and ROS accumulation, which might affect the intracellular availability of important nutrients and oxygen-derived free radicals. Nrf2 buffers labile iron by modulating FPN1 expression exported from the cytosol. The transcriptional regulatory control of miRNAs can serve as an integral molecular mechanism during monocyte differentiation^45^. Our results clearly demonstrated that, in FPN1-knockout myeloma cells, Nrf2 partially restored the intracellular iron and ROS levels in response to iron deprivation, following brusatol treatment. The importance of low FPN1 expression in myeloma cells led us elucidate the mechanisms whereby miR-17-5p modulates Nrf2-mediated, FPN1-dependent regulation of iron metabolism in MM. Compared with other key roles in iron homeostasis (such as in activating CYBRD1), we found that TFRC, and IRP2 upregulation, and FTH1 downregulation were significantly induced after incubation with the miR-17-5p inhibitor in a degree of iron deficiency. Previously, FPN1 overexpression markedly delayed tumor progression by decreasing the tumor burden and prolonging survival during the iron-deficient state, whereas iron administration accelerated tumor progression in vivo^35^. Because cellular iron homeostasis is regulated by a sophisticated system, we believe it will be very interesting to explore the physiological role of FPN1 in iron metabolism, using conditional-knockout mouse models in a follow-up study.

**Fig. 6:**
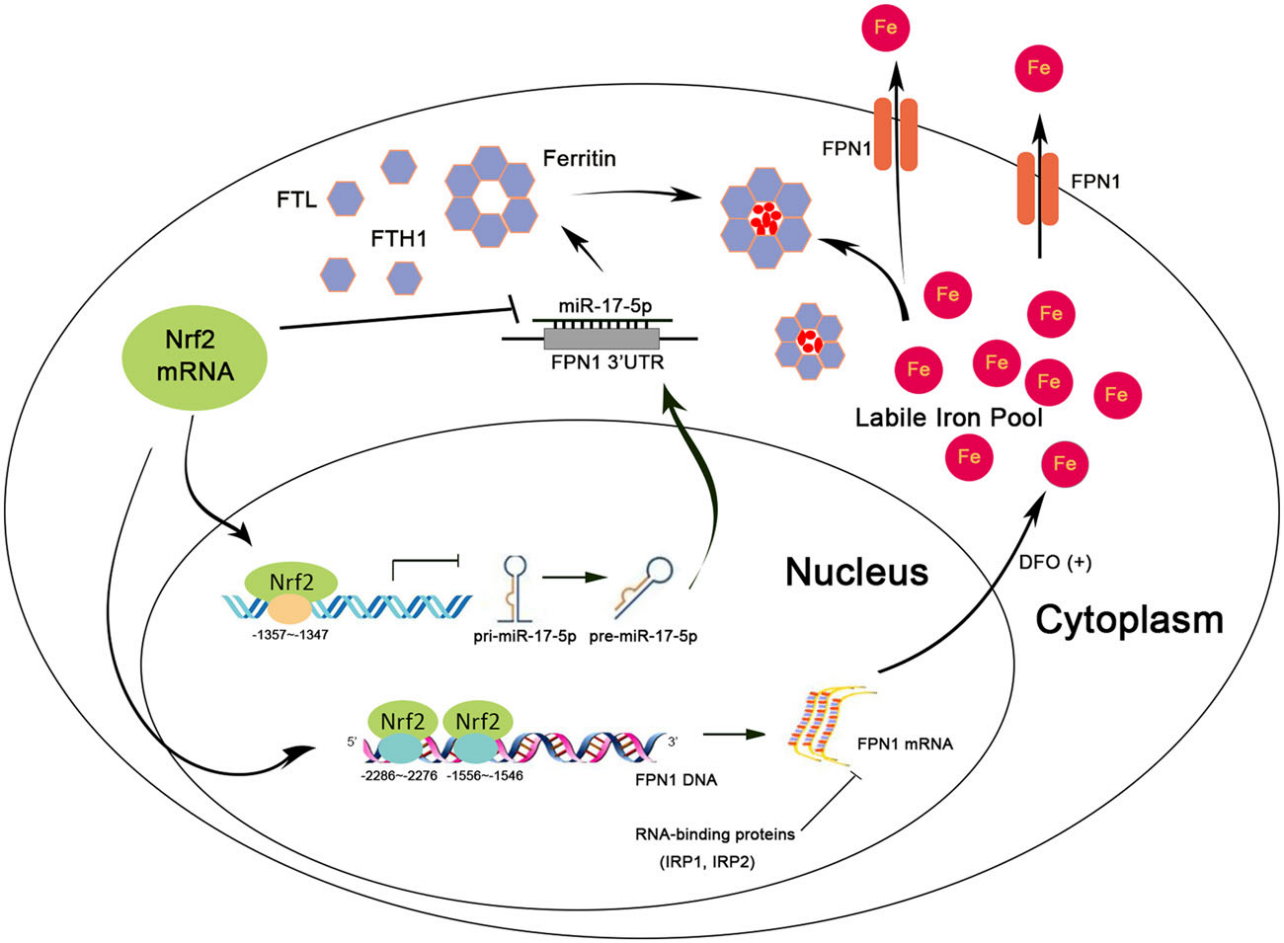
A schematic diagram illustrating the mechanism underlying the tumor suppressor role of FPN1 in multiple myeloma. Nrf2 modulates intracellular iron level by regulating the expression of the iron exporter FPN1. The transcription factor Nrf2 negatively transactivate miR-17-5p expression. Both IRP-mediated 5′UTR regulation and miR-17-5p-mediated FPN 3′UTR regulation contribute to overall post-transcriptional regulation of FPN1

In conclusion, we found that Nrf2 directly transactivated FPN1 expression or inhibited miR-17-5p to target intracellular FPN1 (Fig. 6), which led to the further understanding that iron metabolism is mediated by the transcriptional regulation of FPN1. These studies identify new roles of both miRNAs and IRPs as iron-responsive, post-transcriptional regulators of iron regulation in MM disease progression. While much work remains before in vitro data can be translated to clinical applications, the results of this study highlight the utility of FPN1 in terms of both prognostic value and the potential importance in personalizing cancer therapy.

## Materials and methods

### Gene expression

Gene Expression Omnibus data sets (GSE2658, GSE5900, and GSE19784) were used for gene-expression analyses^32–34^. Probe 223044_at was used to detect the FPN1 transcript with the Affymetrix Human Genome U133 Plus 2.0 Array.

### Reagents and antibodies

DFO (catalog number D9533) and FeCl_3_ (catalog number 157740) were purchased from Sigma-Aldrich (St. Louis, MO, USA). Brusatol, a Nrf2 inhibitor, was purchased from Rongbai biological technology, Co., Ltd. (Shanghai, China). The Cell Counting Kit-8 was purchased from Dojindo (Kumamoto, Japan) and the BD Pharmingen™ Annexin V/propidium iodide (PI) Apoptosis Detection Kit was obtained from BD Biosciences (Franklin Lakes, NJ, USA). The antibodies used in this study are listed in Supplementary Table 1.

### Cells and cell culture

Cell lines were authenticated by Short Tandem Repeat profiling (American Type Culture Collection [ATCC], Manassas, VA, USA). The human MM cell lines (ARP1 and OCI-MY5) were cultured in RPMI-1640 medium (Gibco, Thermo Fisher Scientific, Inc., Waltham, MA, USA), supplemented with 10% fetal bovine serum (FBS; Gibco, Thermo Fisher Scientific, Inc.) and 1% penicillin–streptomycin (Gibco, Thermo Fisher Scientific, Inc.). Human epithelial kidney 293T (HEK293T) cells were maintained in Dulbecco’s modified Eagle medium with 10% FBS and penicillin (100 U/mL)/streptomycin (100 μg/mL). ARP1 and OCI-MY5 cells were gifts from Fenghuang Zhan (Department of Internal Medicine, University of Iowa, Iowa City, IA, USA). HEK293T cells were purchased from the ATCC. All cells were cultured at 37 °C in a humidified atmosphere containing 5% CO_2_ and 95% air.

### Statistical analysis

Data are presented as the mean ± SD of three independent experiments. Statistical analysis was conducted using an unpaired Student’s *t*-test or one-way analysis of variance followed by least-significant difference testing for multiple comparisons. Pearson’s correlation analysis was used to calculate the regression and correlation between two groups. All statistical analyses were performed using SPSS statistical-analysis software, version 20.0 (IBM Corp., Armonk, NY, USA). *p* < 0.05 was considered to reflect a statistically significant difference.

## Supplementary information

Revised Supplementary Material

Supplementary File
