# Supplementary material for: Ferroportin downregulation promotes cell proliferation by modulating the Nrf2–miR-17-5p axis in multiple myeloma
Source: Cell Death Dis. 2019 Aug 19;10(9):624. doi: 10.1038/s41419-019-1854-0 (PMC6698482; doi:10.1038/s41419-019-1854-0)
Supplement: Supplementary file 1 — Revised Supplementary Material [file 41419_2019_1854_MOESM1_ESM.doc]

**Supplementary Materials and Methods**

**Cell transfection**

MiR-17-5p mimic, inhibitor and the negative controls were synthesized and purchased from Shanghai GenePharma Co. Ltd (Shanghai, China). All RNA transfections were performed at 50 nM using Lipofectamine RNAiMAX Reagent (Invitrogen, Carlsbad, CA, USA). While, 2 μg of plasmid DNA were transfected using the Lipofectamine 3000 reagent (Invitrogen). The transfection efficiency were examined by quantitative real-time polymerase chain reaction (qRT-PCR) and Western blot. The sequences are shown in Supplementary Table .

**Cell viability and apoptosis assays**

Cell viability was assessed by Cell Counting Kit-8 (Yeasen, Shanghai, China) colorimetric assay. MM cell lines were seeded in 96-well plates at a density of 2.0 × 10^4^ cells per well with different treatment for 48 h in the incubator. After the incubation, 10 µl of CCK-8 solution was added to each well, and the cells were further incubated for an additional 2 h at 37 °C. The absorbance was measured at 450 nm using a microplate reader. To examine apoptotic cell death, MM cells were washed with ice-cold phosphate buffered saline (PBS) twice and stained with Annexin V-FITC/PI dye protected from light, and analyzed by a BD FACSCanto II flow cytometer (BD Bioscience). Apoptotic cells were identified as both Annexin V ^+^ /PI − staining (early apoptosis) and Annexin V ^+^ /PI ^+^ staining (late apoptosis).

**Cell cycle analysis**

Harvested cells were fixed with 70% ice-cold ethanol overnight at -20 °C. After washing with cold PBS twice, cells were incubated with 500 μ L of staining solution (10 μg/mL PI and 5U/mL RNaseA ) for 15 min at room temperature and analyzed using flow cytometry. Results were analyzed by ModFitLT 3.2 (Verity Software House, Inc., Topsham, ME, USA).

**Soft-agar clonogenicity assay**

1.0×10^4^ transduced myeloma cells were mixed with 0.5 mL 0.33% agar/RPMI1640 media containing 10% FBS and layered on top of the base layer of 0.5% agar, and plated into 12-well culture plates^1^. Cells were incubated (37°C, 5% CO_2_ ) for approximately 2 weeks. The colonies were stained with 0.005% crystal violet and pictured using a digital camera.

**Quantitative real-time PCR**

Total RNA was extracted using TRIzol reagent (Invitrogen) according to the manufacture’s protocol. cDNA was reverse-transcribed from 1 μg of total RNA with oligo (dT) primers using the PrimeScript^TM^ RT Reagent Kit (Takara). Quantitative real-time PCR was performed in 96-well plates by using SYBR Green PCR Master Mix (Kapa biosystems) and Applied Biosystems 7900HT Fast Real-Time PCR System (Thermo Fisher Scientific). The gene expression levels were determined using the 2^-ΔΔCt^ algorithm with U6 or GAPDH as an internal control. Specific primers for each gene transcript are listed in Supplementary Table .

**Western Blotting**

Cells were treated, harvested, lysed on ice for 30 min in lysis buffer [100 mM Tris-HCl (pH 6.8), 4% SDS, 20% glycerol] and protein concentration was measured using the bicinchoninic acid method. The protein samples were separated by SDS-PAGE in 8-12% polyacrylamide gels, transferred to nitrocellulose and blocked with 5% non-fat dry milk or 5% bovine serum albumin at room temperature for 1 h, and then were probed with the relevant primary antibodies overnight at 4℃. Subsequently, the membranes were probed with horseradish peroxidase (HRP)-conjugated secondary antibodies for 1 h at room temperature. Protein bands were visualized using the Odyssey two-color infrared laser imaging system (LI-COR Biosciences).

**Iron concentration assay**

Cells (2 × 10 ^5^ cells/mL) were plated into 6-well plates and treated with different compouds (DFO or brusatol) for 48 h in the incubator. Cells were harvested, washed and transferred to 96-well plates for measuring OD at 590nm ^2^. Iron concentration assay was determined by the QuantiChrom^TM^ Iron Assay Kit (DIFE-250, BioAssays Systems, CA, USA) according to the manufacture’s instructions.

**Reactive oxygen species (ROS) levels**

Cells were treated with different reagent for 48 h in the incubator. After treatment, cells were incubated with 10 μM carboxy-2’7’-dichlorodihydrofluorescein diacetate (H_2_DCFDA) (Life Technologies)in serum-free cell culture medium at 37 °C for 30 min to assess H_2_O_2_-mediated oxidation to 2’7’-dichlorodihydrofluorescein (DCF) as measured at an emission wavelength of 525 nm and an excitation wavelength of 488 nm.

**Plasmid Construction and Lentivirus preparation**

For FPN1 overexpression, the complete human FPN1 CDS fragment was amplified with primer sequences and inserted into the XbaI-EcoRI restriction sites of the pCDH-CMV-MCS-EF1-GFP vector (Promega). While, the Nrf2 overexpressed plasmid was also constructed as described previously. Lentivirus was produced in HEK293T packaging cells using psPAX2 and vsv-g helper plasmid. The lentiviral particles for miR-17-5p overexpression and inhibition constructs were packaged and purchased from GeneChem (Shanghai, China). ARP1 and OCI-MY5 were infected with recombinant lentivirus transducing units plus 5μg/ml Polybrene (Sigma, Natick, MA, USA). Moreover, FPN1 and Nrf2 knockout cell lines were generated via CRISPR/Cas9 system. First, sgRNA sequences were selected to target the FPN1 gene via CRISPR designer at <http://crispr.mit.edu/>. Then, the LentiCRISPRv2 plasmid for sgRNAs, as well as Cas9 expression, was co-transfected with viral packaging plasmids (psPAX2 and pMD2G) into HEK293T cells. The pGL3-miR-17-5p was constructed and the fragments were connected to pGL3-Bsaic vector at the XhoI and HindIII sites. The pGL3-miR-17-mut was generated by site-directed mutagenesis using pGL3-miR-17-5p as template. Primers are listed in Supplementary Table. All plasmid constructs were confirmed by DNA sequencing analyses.

**Chromatin Immunoprecipitation**

ChIP assay was performed using One-Day Chromatin Immunoprecipitation Kits (17-10085/10086, Millipore, Darmstadt, Germany). Briefly, 1.0×10^7^ cells were crosslinked with 1% formaldehyde for 10 min at room temperature, washed in PBS, and then resuspended in ChIP nuclear lysis buffer. The cross-linked DNA were sonicated to create appropriately sized chromatin fragments less than 1000 base pairs (verified by agarose gel analysis). After clarifying lysates by centrifugation, supernatants were incubated overnight with antibodies at 4℃ and an aliquot of the pre-clear chromatin was used as an input, followed by incubation with fully resuspended Protein A/G magnetic beads. After washing beads, immune complex were eluted by heating at 62℃ with shaking. Crosslinking of protein-DNA complex were reversed by incubation 2h. After purification, immunoprecipitated DNA was analysed by PCR. Primer sequences designed by the putative binding sites are listed in Supplementary Table.

**Dual-luciferase reporter assay**

The 3’UTR sequence of human FPN1 gene was amplified by PCR from human liver genomic DNA, and cloned into the XhoI and NotI sites downstream of the luciferase-expressing vector psiCHECK2 (Promega). The mutant 3’UTR clone was constructed by introducing several mutations in miR-17-5p binding site using QuickChange Ⅱ XL Site-Directed Mutagenesis Kit (Agilent Technologies) according to kit manual, called Wt-FPN1 3’UTR and Mut- FPN1 3’UTR. Subsequently, HEK293T cells were transiently co-transfected with miR-17-5p mimic or miR-NC and FPN1 reporter plasmid (wild-type or mutant) by using RNA iMAX reagent (Invitrogen). In addition, co-transfection was performed with pGL3-Basic , pGL3-FPN1 and truncated bodies reporter plasmid, pGL3-miR-17-5p and mutant plasmid, pCDH-CMV-MCS-EF1-GFP, pCDH-CMV-MCS-EF1-GFP-Nrf2 and Renilla plasmid. Cells harvested were prepared 48h after transfection, and relative firefly and Renilla activities were measured using the Dual-Luciferase Reporter Assay System (Promega) following the manufacture’s instruction. The relative luciferase activity of each sample were analyzed as the activity of firefly relative to Renilla.

**Xenograft**

Female BALB/c nude mice (4-6 week of age and weighting 17-20 g) were purchased from Shanghai Laboratory Animal Center (SLAC, Shanghai, China). All mice were housed in the animal care facility, under a 12‑hour light/dark cycle at 24˚C, and they were fed with a standard diet and acidified water ad libitum. Next, human ARP1 cells (2.5× 10 ^6^) that were transduced with miR-17-5p or miR-NC vectors or anti-miR-17-5p were suspended in 100 µl serum-free culture medium and were subcutaneously injected into the upper flank of nude mice. In addition, ARP1-FPN1^OE^ cells, co-transfected with miR-17-5p-overexpressing , were also enrolled in this study. The tumor volume was monitored every 3 days and calculated as (length × width ^2^ ) x 0.5. After 3 weeks, the mice were sacrificed by CO_2_ asphyxiation and the tumors were obtained and imaged. The protocols for animal experiments were approved by the institutional review board of the Shanghai Tenth People's Hospital (ID: SYXK 2011-0111).

**References:**

1. Yang, Y. *et al.*, NEK2 mediates ALDH1A1-dependent drug resistance in multiple myeloma. *ONCOTARGET* **5** 11986 (2014).
2. Habel, M. E. & Jung, D., c-Myc over-expression in Ramos Burkitt's lymphoma cell line predisposes to iron homeostasis disruption in vitro. *Biochem Biophys Res Commun* **341** 1309 (2006).

**Supplementary Table**

**Supplementary Table 1. Antibodies**

| **Antibodies** | **Sources** | **Catlog #** | **Applications** |
| --- | --- | --- | --- |
| FPN1 | Novus biologicals | NBP1–21502 | WB |
| Nrf2 | Abcam | ab62352 | ChIP, WB |
| Cleaved Caspase 3 | Cell signaling technology | 9661 | WB |
| Cleaved Caspase 8 | Cell signaling technology | 9496 | WB |
| PARP | Cell signaling technology | 9542 | WB |
| Cyclin D1 | Abcam | ab134175 | WB |
| CDK4 | Abcam | ab108357 | WB |
| CDK6 | Abcam | ab124821 | WB |
| GAPDH | Cell signaling technology | 2118 | WB |
| FLAG-Tag | Cell signaling technology | 8146 | WB |
| Actin-HRP | Santa Cruz Biotechnology | sc-1616 | WB |
| rabbit IgG-HRP | Cell signaling technology | 7074 | WB (2nd Ab) |
| mouse IgG-HRP | Cell signaling technology | 7076 | WB (2nd Ab) |
| Normal rabbit IgG | Cell signaling technology | 2729 | ChIP (control) |

WB, western blot. ChIP, chromatin immunoprecipitation. HRP, horseradish peroxidase.

**Supplementary Table 2.** RNA oligonucleotides

| **Target regions** | **Directions** | **Sequences (5' to 3')** |
| --- | --- | --- |
| miR-17 mimic | sense | CAAAGUGCUUACAGUGCAGGUAG |
|  | antisense | ACCUGCACUGUAAGCACUUUGUU |
| negative control mimic | sense | UUCUCCGAACGUGUCACGUTT |
|  | antisense | ACGUGACACGUUCGGAGAATT |
| miR-17 inhibitor | antisense | CUACCUGCACUGUAAGCACUUUG |
| negative control inhibitor | antisense | CAGUACUUUUGUGUAGUACAA |

**Supplementary Table 3.** FPN1, Nrf2 oe/sgRNA sequences

| **Target genes** | **Directions** | **Sequences (5' to 3')** |
| --- | --- | --- |
| FPN1 oe | Forward | GCTCTAGAATGACCAGGGCGGGAGATC |
|  | Reverse | CCGGAATTCTCAAACAACAGATGTATTTG |
| FPN1 sgRNA#1 | Forward | CACCGCGTAGACTGCTGTCAAAAGG |
|  | Reverse | AAACCCTTTTGACAGCAGTCTACGC |
| FPN1 sgRNA#2 | Forward | CACCGCTGAGTGTAGGCGTACCCTG |
|  | Reverse | AAACCAGGGTACGCCTACACTCAGC |
| FPN1 sgRNA#3 | Forward | CACCGCTGTGTCACAGTTAAATCAA |
|  | Reverse | AAACTTGATTTAACTGTGACACAGC |
| Nrf2 oe | Forward | TGCTCTAGAATGGATTTGATTGACATACT |
|  | Reverse | CGCGGATCCCTAGTTTTTCTTAACATCTG |
| Nrf2 sgRNA#1 | Forward | CACCGCGTTGAAGTCAACAACAGGG |
|  | Reverse | AAACCCCTGTTGTTGACTTCAACGC |
| Nrf2 sgRNA#2 | Forward | CACCGCATTAATTCGGGATATACGT |
|  | Reverse | AAACACGTATATCCCGAATTAATGC |
| Nrf2 sgRNA#3 | Forward | CACCGTATTTGACTTCAGTCAGCGA |
|  | Reverse | AAACTCGCTGACTGAAGTCAAATAC |

**Supplementary Table 4.** Primers for realtime PCR

| **Target genes** | **Directions** | **Sequences (5' to 3')** |
| --- | --- | --- |
| TFRC | Forward | ACCATTGTCATATACCCGGTTCA |
|  | Reverse | CAATAGCCCAAGTAGCCAATCAT |
| FTL | Forward | CAGCCTGGTCAATTTGTACCT |
|  | Reverse | GCCAATTCGCGGAAGAAGTG |
| FTH1 | Forward | CCCCCATTTGTGTGACTTCAT |
|  | Reverse | GCCCGAGGCTTAGCTTTCATT |
| IRP2 | Forward | TCGATGTATCTAAACTTGGCACC |
|  | Reverse | GCCATCACAATTTCGTACAGCAG |
| Nrf2 | Forward | GAGAGCCCAGTCTTCATTGC |
|  | Reverse | TGCTCAATGTCCTGTTGCAT |
| CYBRD1 | Forward | GCTCCGCTTTCTCTCCGAG |
|  | Reverse | TGTCAATCCCATAAGTGCTGTTG |
| GAPDH | Forward | GGAGCGAGATCCCTCCAAAAT |
|  | Reverse | GGCTGTTGTCATACTTCTCATGG |
| U6 | Forward | CTCGCTTCGGCAGCACA |
|  | Reverse | AACGCTTCACGAATTTGCGT |
| FPN1 | Forward | CTACTTGGGGAGATCGGATGT |
|  | Reverse | CTGGGCCACTTTAAGTCTAGC |
| miR-17-5p | Forward | GCCGGCGTCAGAATAATGTCAAAGTGC |
|  | Reverse | CACCATAATGCTACAAGTGCCTTCACTGC |

**Supplementary Table 5.** Primers for ChIP assay

| **Target regions** | **Directions** | **Sequences (5' to 3')** |
| --- | --- | --- |
| FPN1 TSS Ⅰ | Forward | GGGATTTTTAAAAAGGTGGT |
|  | Reverse | AGTTTACAAAAAATAGTAGATACCC |
| FPN1 TSS Ⅱ | Forward | GTTTTCTCAGAAGGAGGAAG |
|  | Reverse | ACTATTCAGAAAGTGCAAAATG |
| FPN1 TSS Ⅲ | Forward | GGGACTCCTTGGTGAC |
|  | Reverse | TAGACACTGTTTAAGAAGTATTT |
| FPN1 TSS Ⅳ | Forward | TATGTAATAATTAATGTAAAGCACTTG |
|  | Reverse | CAACAAGGCACAAAGGA |
| miR-17-5p TSS Ⅰ | Forward | TGCAGCAAAGGGAAAAG |
|  | Reverse | CCGGGATAAAGAGTTGTTT |
| miR-17-5p TSS Ⅱ | Forward | GGACTAAATTGCCTTTAAATGT |
|  | Reverse | TCTTCCAATGGCTGGC |

TSS, transcriptional start site.
