# Supplementary figures and images for: Ferroportin downregulation promotes cell proliferation by modulating the Nrf2–miR-17-5p axis in multiple myeloma
Source: Cell Death Dis. 2019 Aug 19;10(9):624. doi: 10.1038/s41419-019-1854-0 (PMC6698482; doi:10.1038/s41419-019-1854-0)

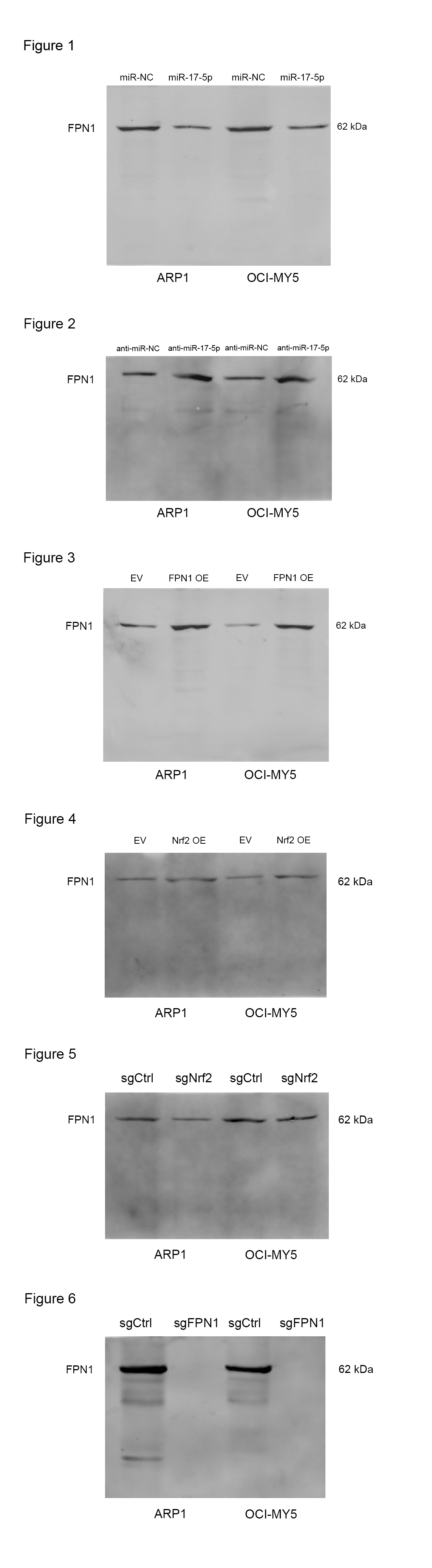

Supplement: Supplementary file 2 — Supplementary File [file 41419_2019_1854_MOESM2_ESM.tif]
